# Supplementary material for: NEK2 inhibition triggers anti-pancreatic cancer immunity by targeting PD-L1
Source: Nat Commun. 2021 Jul 27;12:4536. doi: 10.1038/s41467-021-24769-3 (PMC8316469; doi:10.1038/s41467-021-24769-3)
Supplement: Supplementary file 1 — Supplementary Information [file 41467_2021_24769_MOESM1_ESM.pdf]

Supplementary figures

a

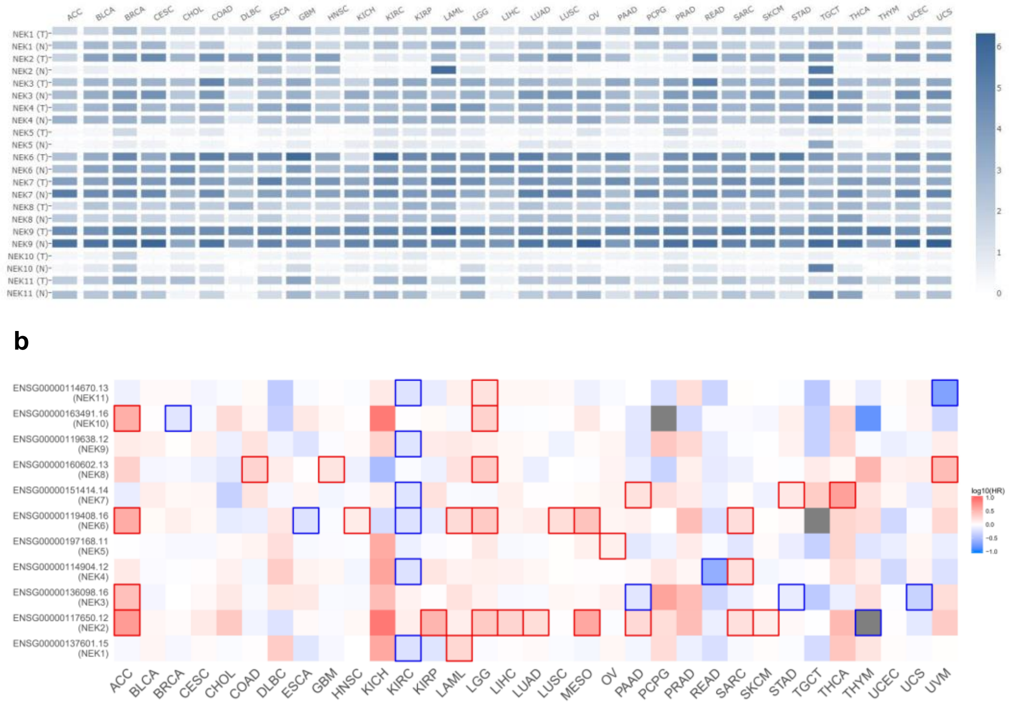

Supplementary Fig. 1: Expression profiles and survival map of NEK family proteins among multiple cancer types.

(**a**) Summary of the expression landscape of the NEK family proteins in multiple cancer types from the TCGA database. (**b**) Survival map of NEK family proteins in multiple cancer types from the TCGA database.

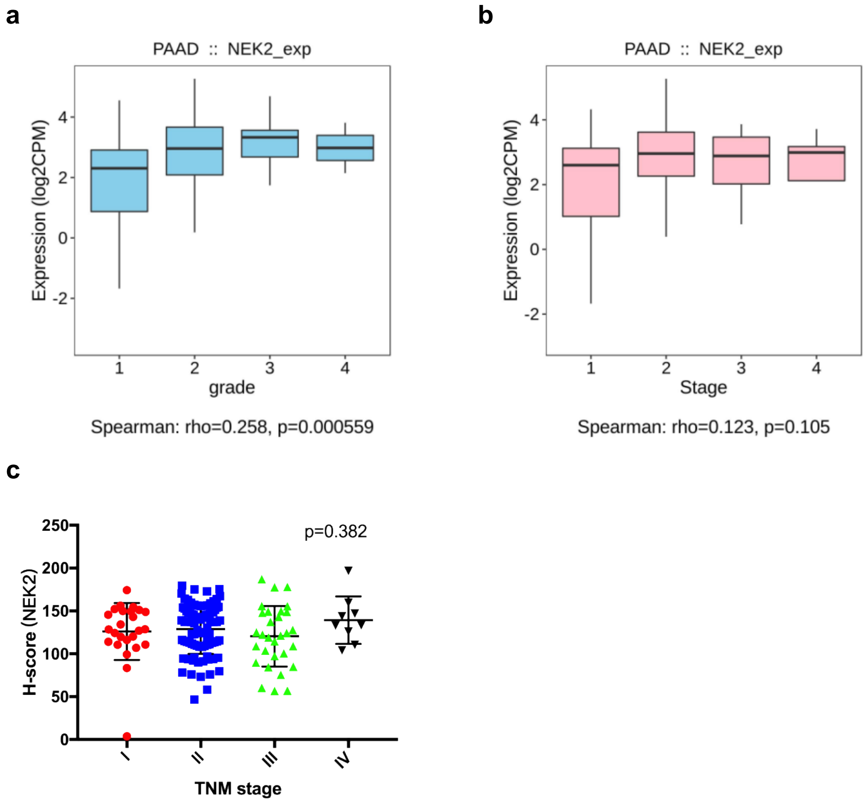

Supplementary Fig. 2: Association between the expression of NEK2 and clinical features.

(**a, b**) Association between the expression of NEK2, tumor stage, and grade in PAAD datasets from TCGA database. (**c**) Association between the expression of NEK2 and tumor stage in PDAC tissue microarray (n (stage I) = 25, n (stage II) =88, n (stage III) = 30, n (stage IV) =9). One-way and repeated-measures analysis of variance (ANOVA) were used to compare multiple groups in **c**. In **a** and **b**, data are represented as boxplots where the middle line is the median; the lower and upper hinges correspond to the first and third quartiles; the upper whisker extends from the hinge to the largest value no further than 1.5 × IQR from the hinge (where IQR is the inter- quartile range); the lower whisker extends from the hinge to the smallest value at most 1.5 × IQR of the hinge, while data beyond the end of the whiskers are outlying points that are plotted individually.

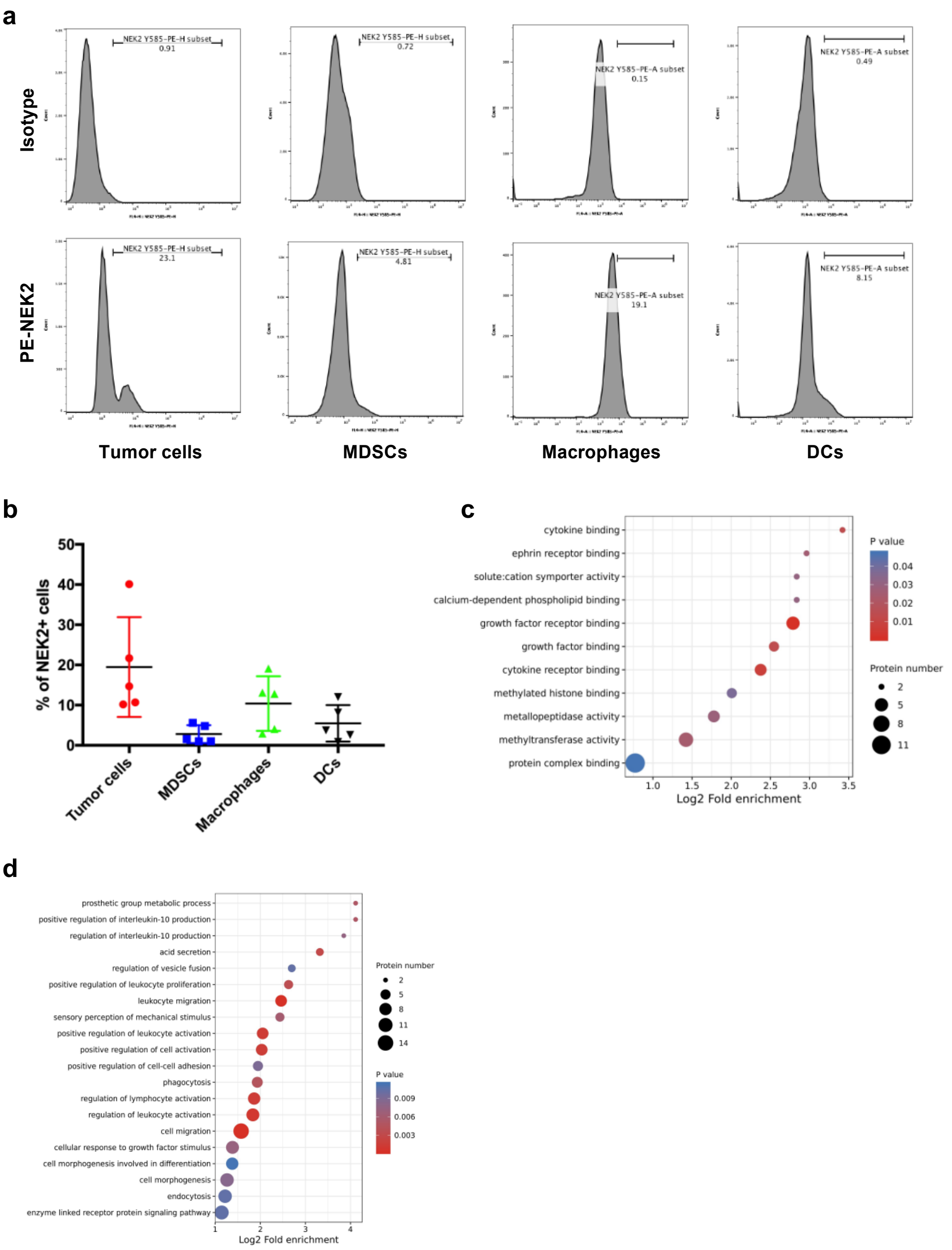

Supplementary Fig. 3: Association between NEK2 expression and the anti-tumor immunity signaling pathway.

(**a, b**) Flow cytometric analysis and statistical analysis of NEK2 expression in immunosuppressive populations in patient PDAC samples (n = 5). (**c, d**) Expression of proteins after *NEK2* overexpression analyzed by GO annotation in quantitative analysis of LC-MS proteomics. Results are presented as means ± SD from one representative experiment in **b**. All data are representative of three independently performed experiments.

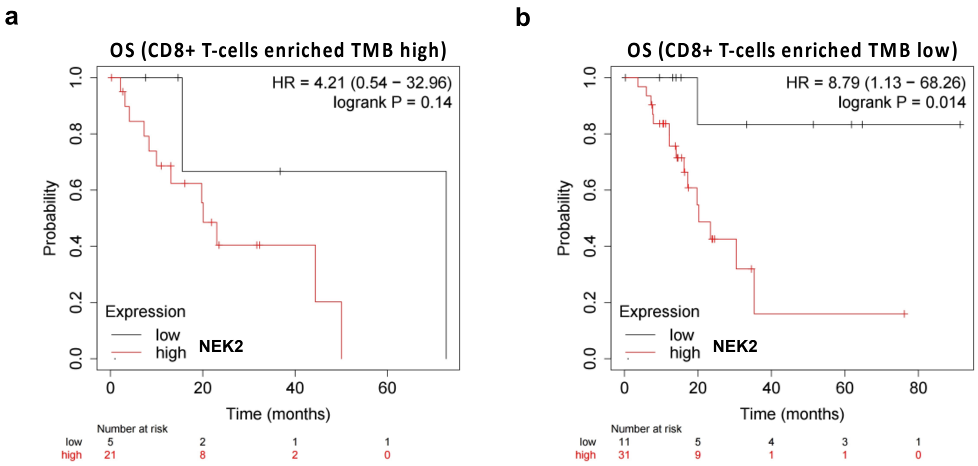

Supplementary Fig. 4: NEK2 correlates with OS in patients with pancreatic cancer with low TMB.

(**a**) Overall survival (OS) of pancreatic cancer patients with enriched numbers of $CD8^+$ T cells, high TMB, and high or low expression of NEK2 (n = 76). (**b**) Overall survival (OS) of pancreatic cancer patients with enriched numbers of $CD8^+$ T cells, low TMB, and high or low expression of NEK2 (n = 101). The Hazard Ratios (HR) and *p*-values by the log-rank (Mantel–Cox) test are indicated in **a** and **b**.

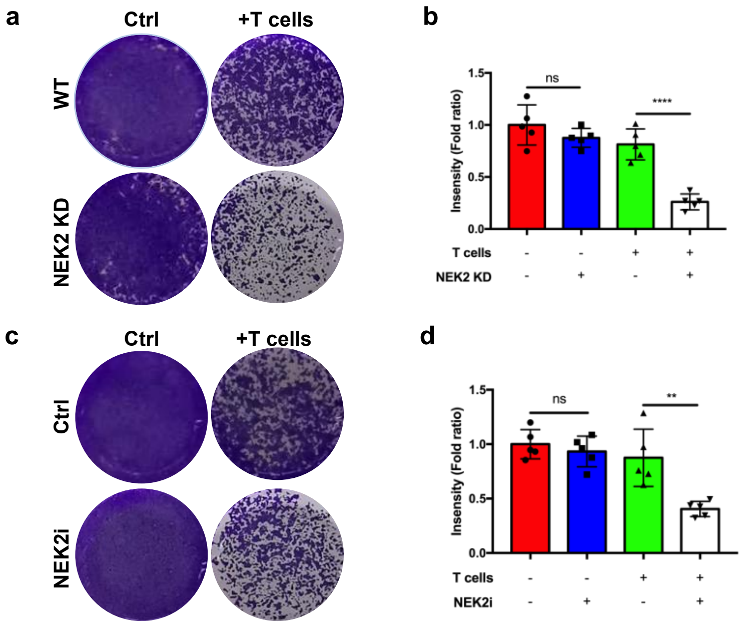

Supplementary Fig. 5: NEK2 inhibits T cell-mediated pancreatic cancer cell-destruction *in vitro*.

(**a-d**) Representative images and statistical analysis of a T cell-mediated cancer cell-destruction assay. KPC cells with *NEK2* knockdown cocultured with activated T cells for 48 h and stained with crystal violet. The ratio of tumor cells to T cells was 1:4. Representative images of live cells are shown (**a**) and further quantified (**b**) (n = 5). KPC cells pretreated with NEK2 inhibitor for 24 h were cocultured with activated T cells for 48 h and stained with crystal violet. The ratio of tumor cells to T cells was 1:4. Representative images of live cells are shown (**c**) and further quantified (**d**) (n = 5). Results are presented as means ± SD of one representative experiment in **b** and **d**. All data are representative of three independently performed experiments. **P* < 0.05, ***P* < 0.01, ****P* < 0.001 using a two-tailed t-test; ns: not significant.

a

A	B	Neither	A Not B	B Not A	Both	Log2	Odds Ratio	p-Value	q-Value Tendency
NEK2	CD274	917	11	10	4	>3	<0.001	<0.001	Co-occurrence

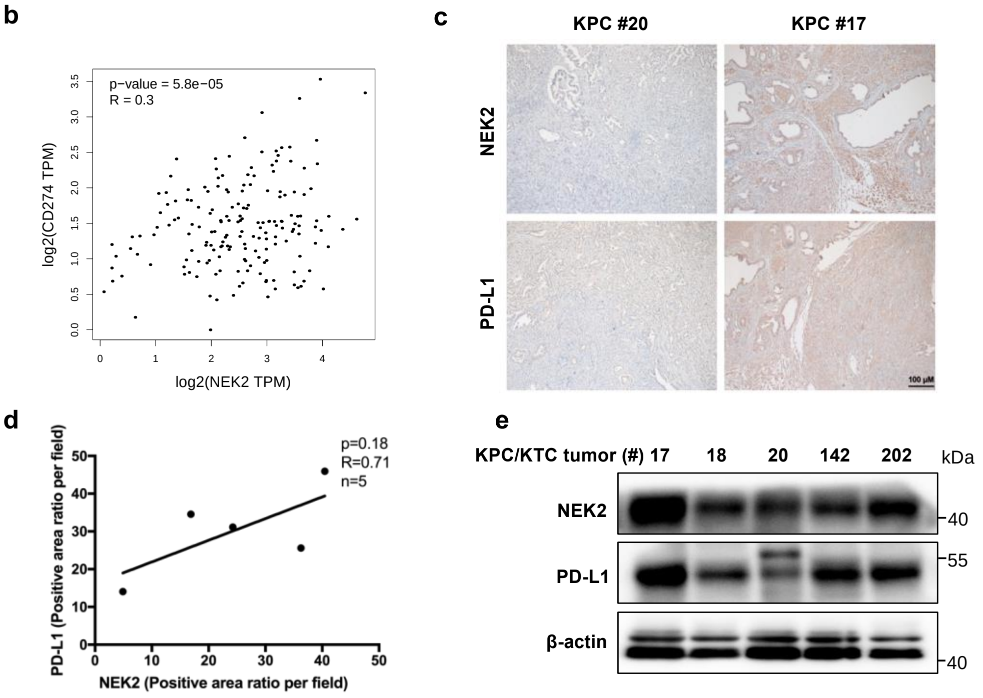

Supplementary Fig. 6: Correlation analysis of NEK2 and PD-L1 in pancreatic cancer.

(**a**) Bioinformatics analysis of mutual exclusivity between NEK2 and PD-L1 in integrated multiple pancreatic datasets from TCGA database, n=179. (**b**) Bioinformatics analysis of the correlation between NEK2 and PD-L1 using PAAD datasets from the TCGA and other databases. (**c, d**) Representative images and statistical analysis of IHC staining of NEK2 and PD-L1 in GEMM autochthonous tumors (KPC or KTC) (n = 5). (**e**) Western blot analysis of NEK2 and PD-L1 expression in pancreatic tissue samples in mice (KPC or KTC) (n = 5). The Spearman correlations and *p*-values by Spearman's test are indicated in **d**.

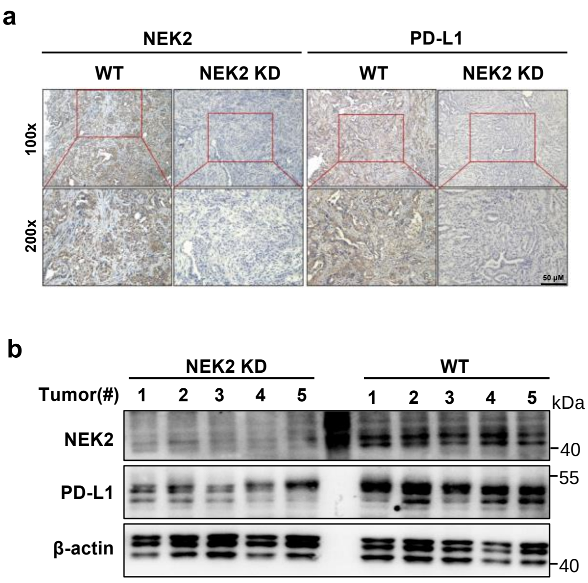

Supplementary Fig. 7: NEK2 positively correlates with PD-L1 in xenograft tumor samples.

(**a, b**) Representative images of IHC staining and Western blotting of NEK2 and PD-L1 in xenograft tumor samples. Scale bars: 100x: 50 µm; 200x: 100 µm.

a

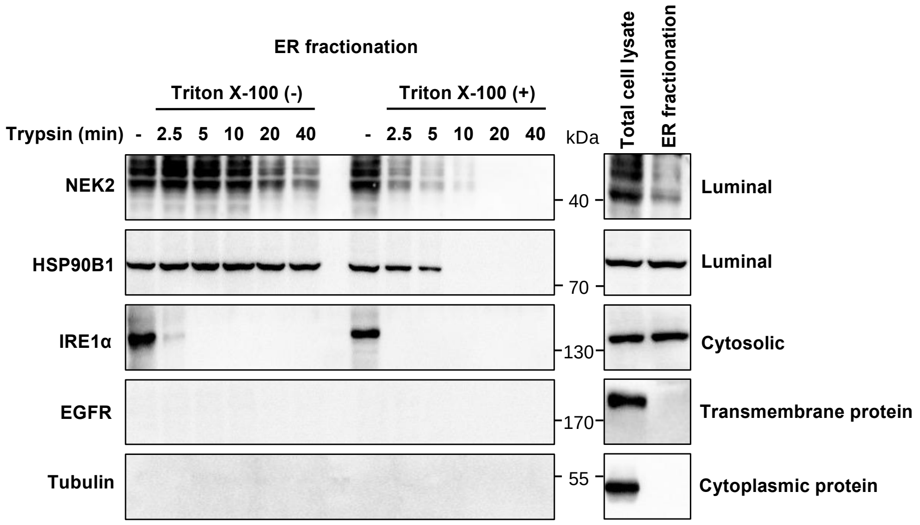

Supplementary Fig. 8: NEK2 regulates PD-L1 stability in the ER.

(**a**) Trypsin digestion of ER fractions with or without permeabilization in KPC cells. Representative image is shown, n = 3 independent experiments.

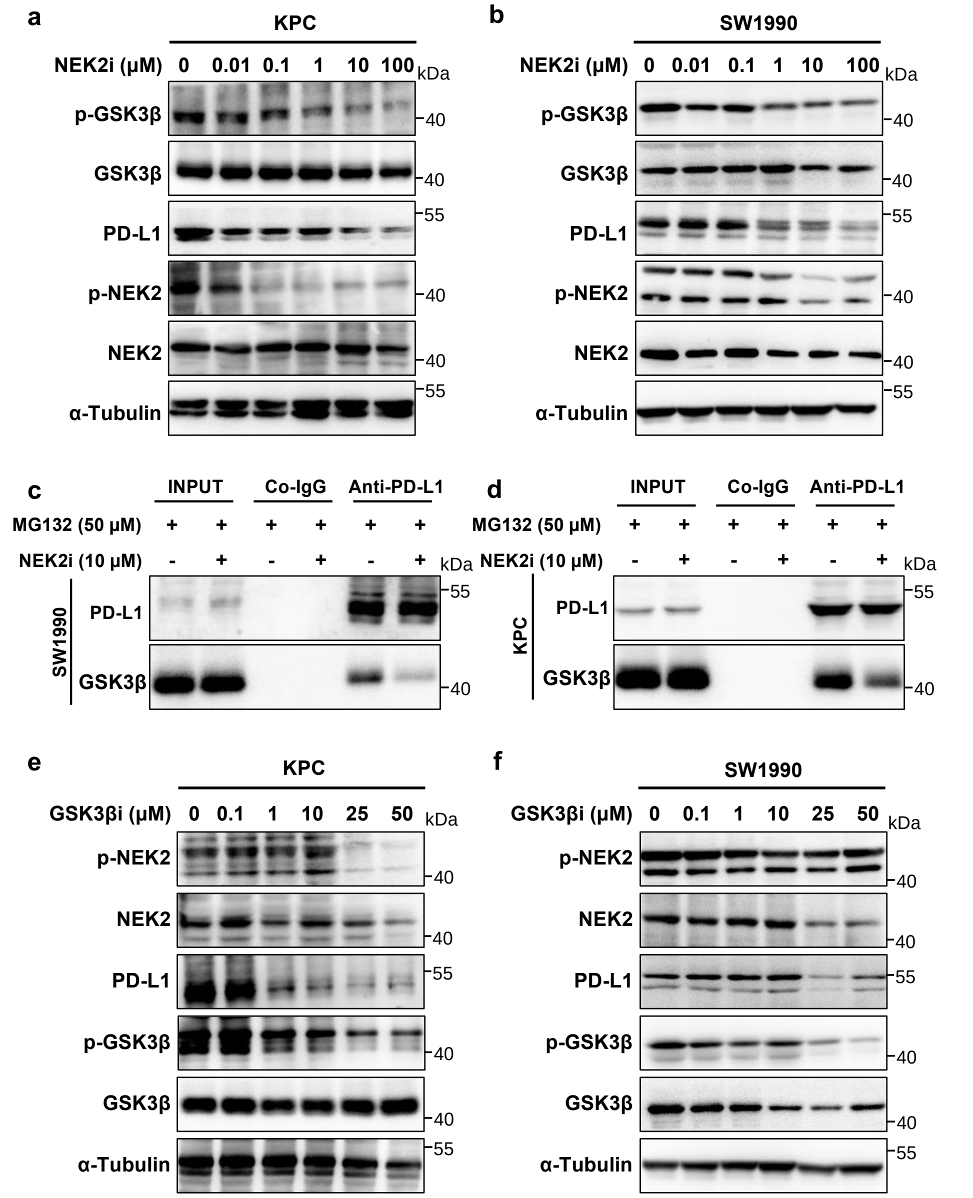

Supplementary Fig. 9: Relationship between the role of NEK2 and GSK3β in pancreatic cancer cells.

(**a, b**) Western blot analysis of p-GSK3β, GSK3β, and PD-L1 in KPC and SW1990 cells treated with NEK2 inhibitor. Representative image is shown, n = 3 independent experiments. (**c, d**) IP analysis of PD-L1 and GSK3β treated with NEK2 inhibitor in SW1990 and KPC cells. Representative image is shown, n = 3 independent experiments. (**e, f**) Western blot analysis of p-NEK2, NEK2, and PD-L1 in KPC and SW1990 cells treated with GSK3β inhibitor. Representative image is shown, n = 3 independent experiments.

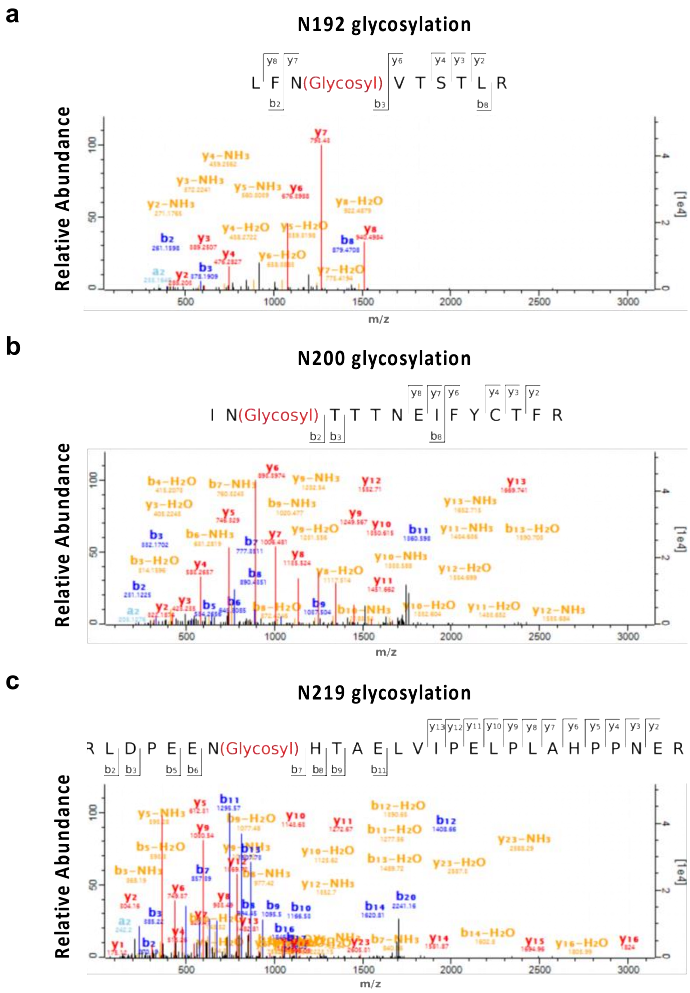

Supplementary Fig. 10: NEK2 overexpression stimulates PD-L1 glycosylation.

(**a-c**) PD-L1 glycosylation at the N192/N200/N219 residues were identified by mass spectrometry analysis.

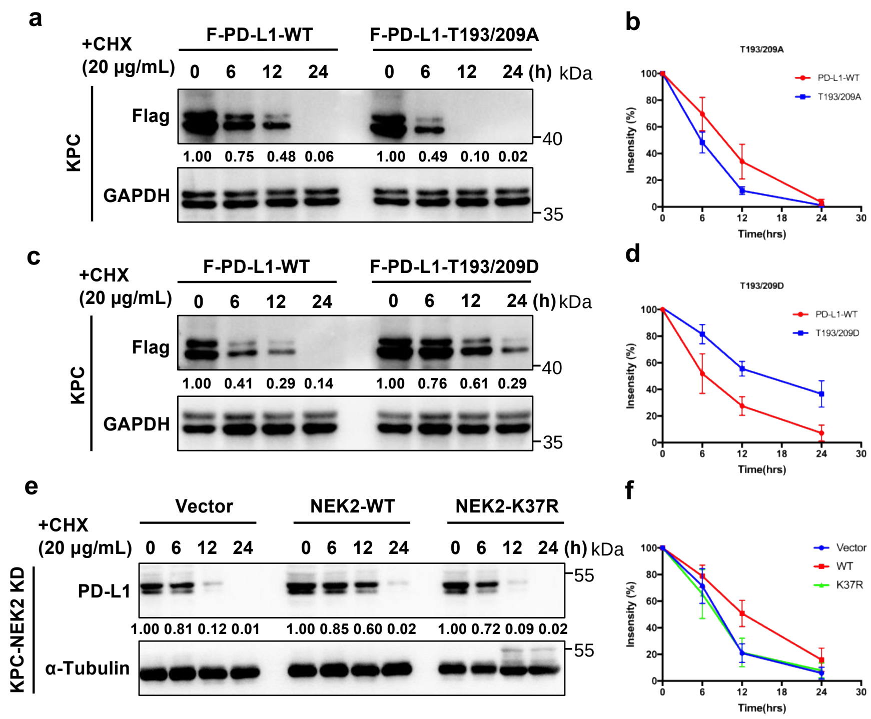

Supplementary Fig. 11: NEK2 phosphorylates PD-L1 by K37 to maintain protein stability.

(**a-d**) Half-life analysis of PD-L1 in Flag-PD-L1 WT and T193/209A or T193/209D KPC cells treated with CHX (20 μg/mL). (**e, f**) Half-life analysis of PD-L1 in WT and K37R KPC cells treated with CHX (20 μg/mL). The intensity of PD-L1 protein expression was quantified using a densitometer. Statistical analysis is based on three independent experiments. Results are presented as means ± SD of one representative experiment in **b**, **d** and **f**. All data are representative of three independently performed experiments. **P* < 0.05, ***P* < 0.01, ****P* < 0.001 using a two-tailed t-test; ns: not significant.

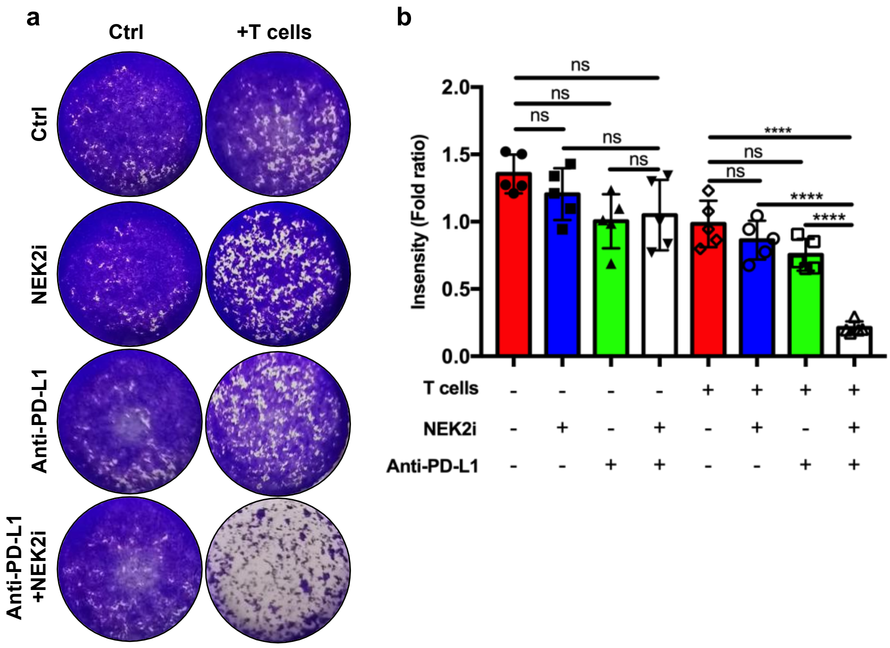

Supplementary Fig. 12: Co-targeting NEK2 and PD-L1 enhances the killing function of T cells *in vitro*.

(**a, b**) Results of a T cell-mediated cancer cell-killing assay. KPC cells pretreated with NEK2 inhibitor for 24 h, with or without PD-L1 blockade, were cocultured with activated T cells for 48 h and stained with crystal violet. The ratio of tumor cells to T cells was 1:4. Representative images of live cells are shown (**a**) and were further quantified (**b**) (n = 5). Results represent means ± SD of one representative experiment in **b**. All data are representative of three independently performed experiments. $*P < 0.05$, $**P < 0.01$, $***P < 0.001$ using a two-tailed t-test; ns: not significant.

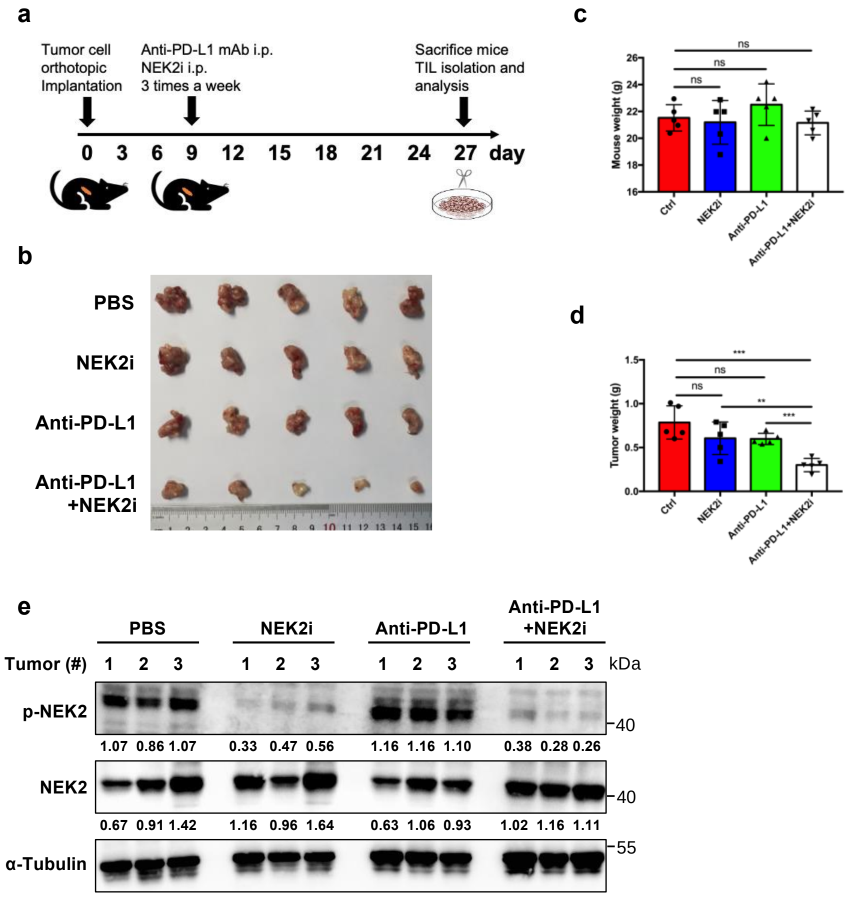

Supplementary Fig. 13: NEK2 inhibition sensitizes PD-L1-targeted pancreatic cancer immunotherapy in an orthotopic model.

(**a**) Schematic protocol of anti-PD-L1 antibody and NEK2 inhibitor combination therapy. (**b**) Representative images displaying tumors harvested from mice bearing KPC cells treated with anti-PD-L1 antibody, NEK2 inhibitor, or their combination (n = 5). (**c**) Tumor growth curve of mice treated with anti-PD-L1 antibody (200 μg/mouse), NEK2 inhibitor (200 μg/mouse), or their combination (n = 5). (**d**) Tumor weight in mice treated with anti-PD-L1 antibody, NEK2 inhibitor, or both (n = 5). (**e**) Western blot analysis of p-NEK2 and NEK2 in tumor samples from an orthotopic model that received PD-L1 antibody in combination with NEK2 inhibitor (n = 3). Results represent means ± SD of one representative experiment in **c** and **d**. All data are representative of three independently performed experiments. *$P < 0.05$, **$P < 0.01$, ***$P < 0.001$ using a two-tailed t-test; ns: not significant.

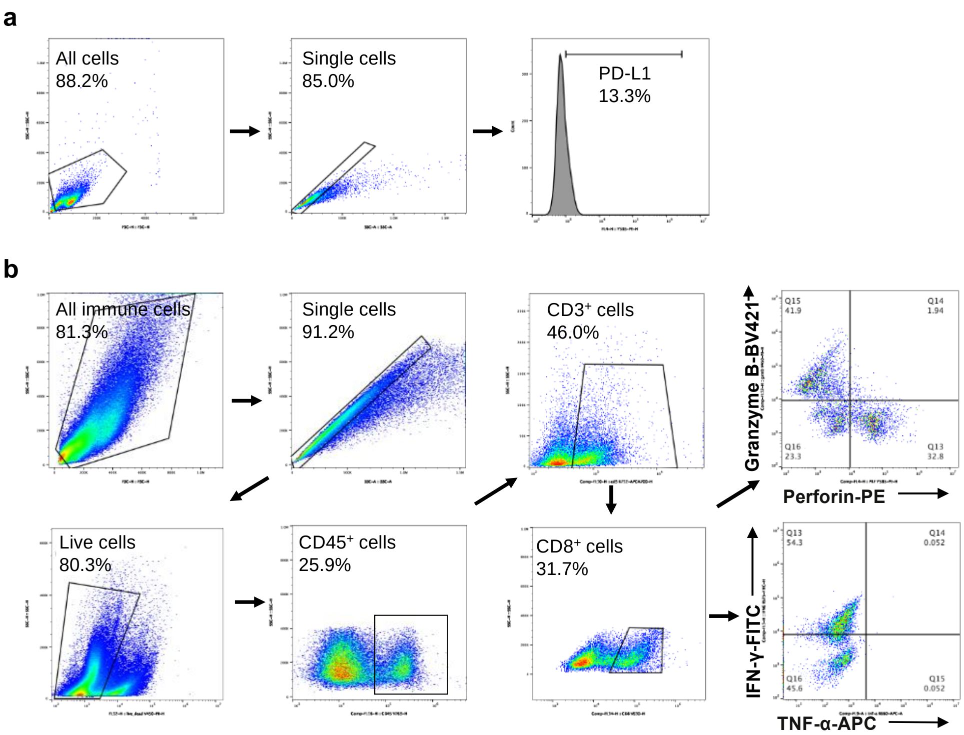

Supplementary Fig. 14: Gating strategies used in FACs analysis for cell lines and mouse-bearing tumors.

(**a**) Gating strategy to analyze the expression of PD-L1 in pancreatic cancer cells. (**b**) Gating strategy to analyze the number and function of $CD8^+$ T cell in mouse-bearing tumors.

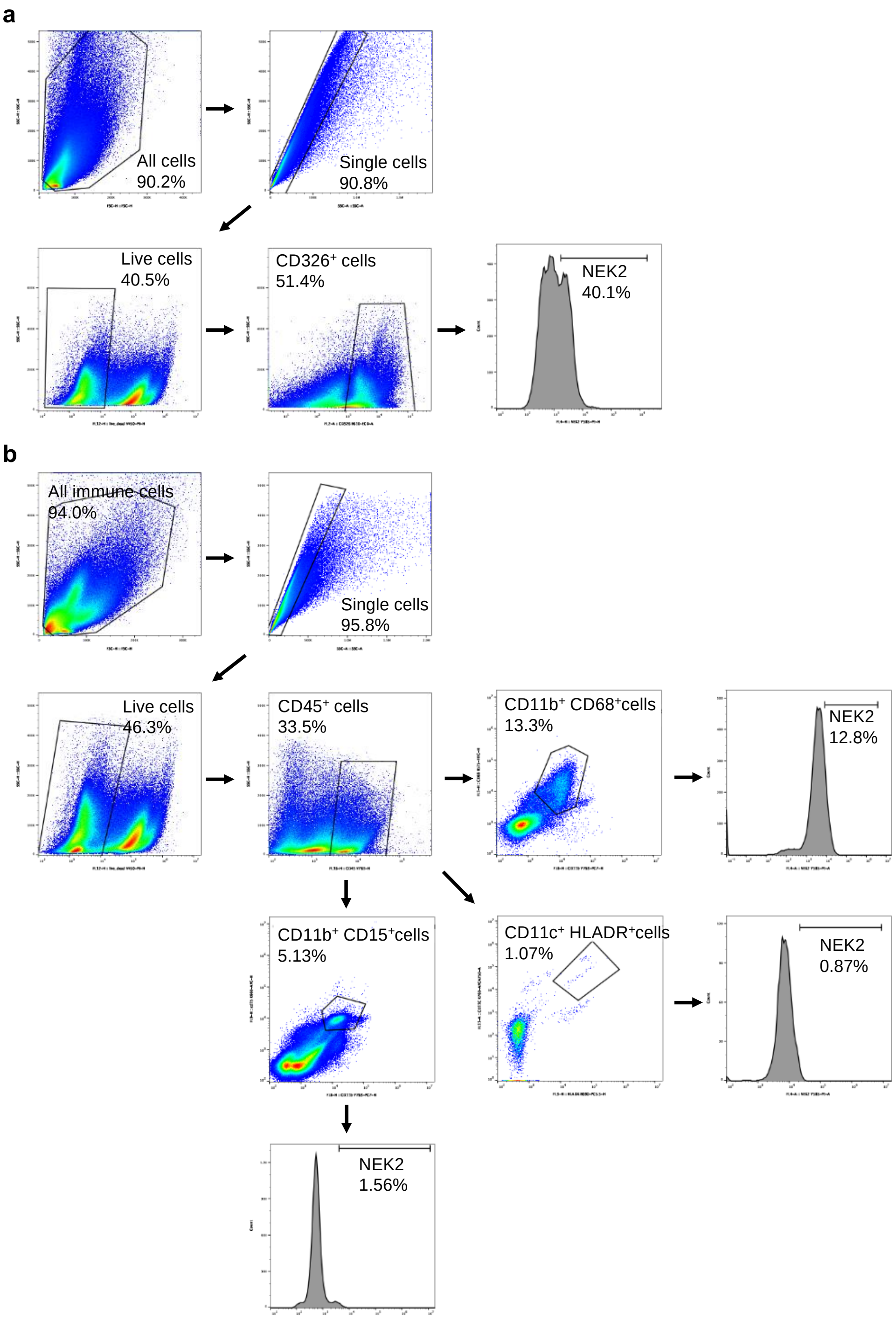

Supplementary Fig. 15: Gating strategies used in FACs analysis for patient PDAC samples.

(**a**) Gating strategy to analyze the expression of NEK2 in pancreatic tumor cells. (**b**) Gating strategy to analyze the expression of NEK2 in immunosuppressive populations (macrophages, DCs and MDSCs) in patient PDAC samples.
