# Supplementary figures and images for: NEK2 inhibition triggers anti-pancreatic cancer immunity by targeting PD-L1
Source: Nat Commun. 2021 Jul 27;12:4536. doi: 10.1038/s41467-021-24769-3 (PMC8316469; doi:10.1038/s41467-021-24769-3)

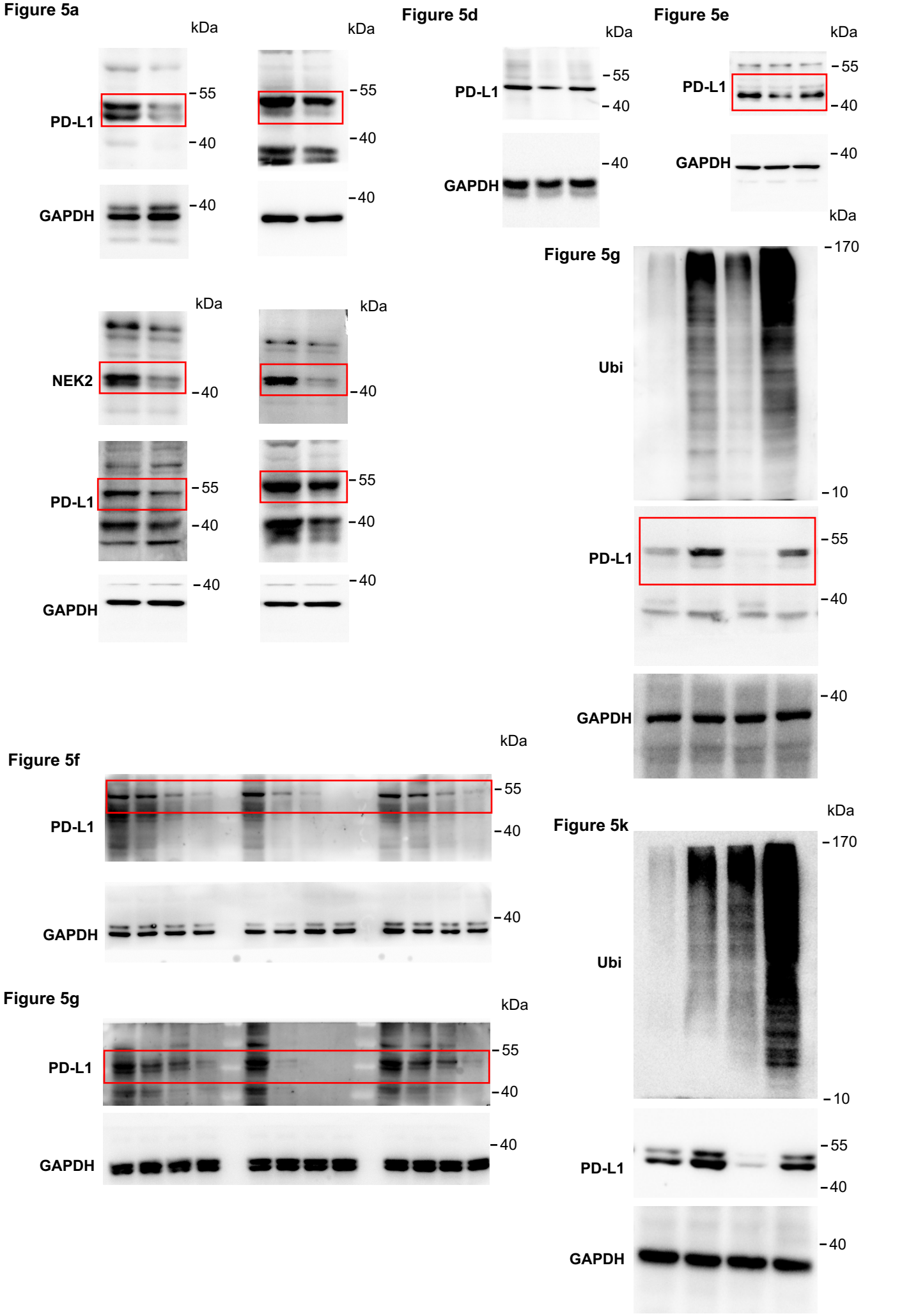

Supplement: Supplementary file 4 — Source data [file 41467_2021_24769_MOESM4_ESM.zip › Source data/Figure 5-uncropped gels.pdf]

**Figure S9a**

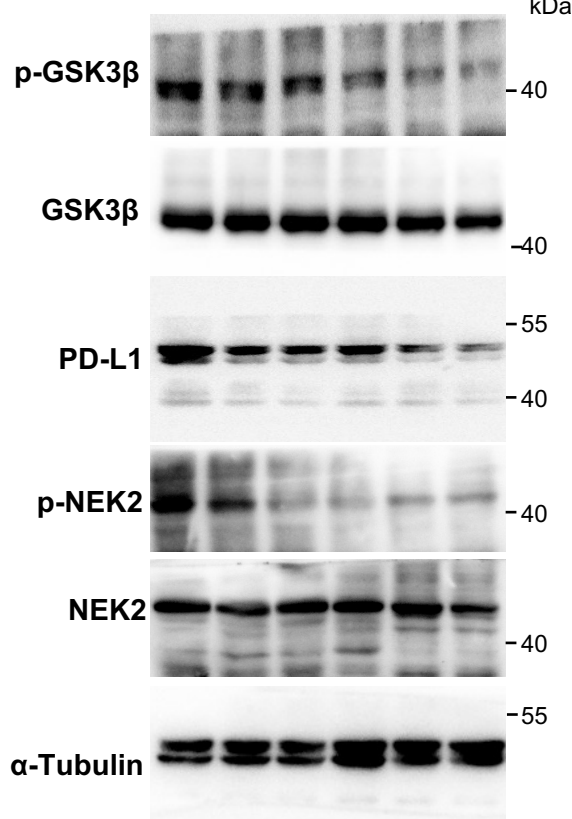

**Figure S9b**

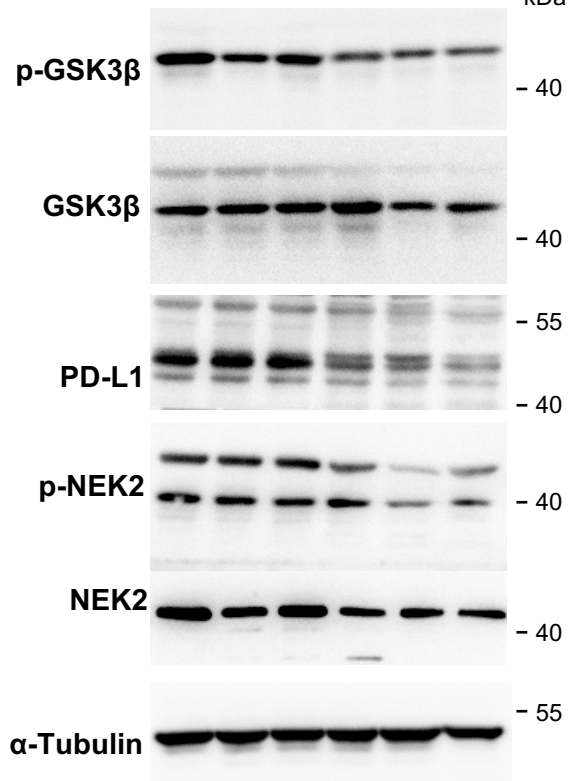

**Figure S9c**

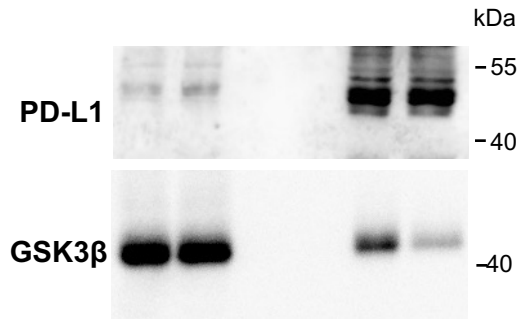

**Figure S9d**

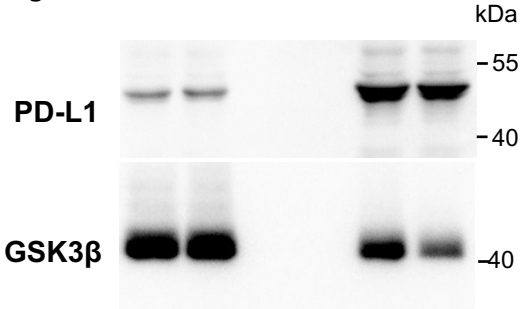

**Figure S9e**

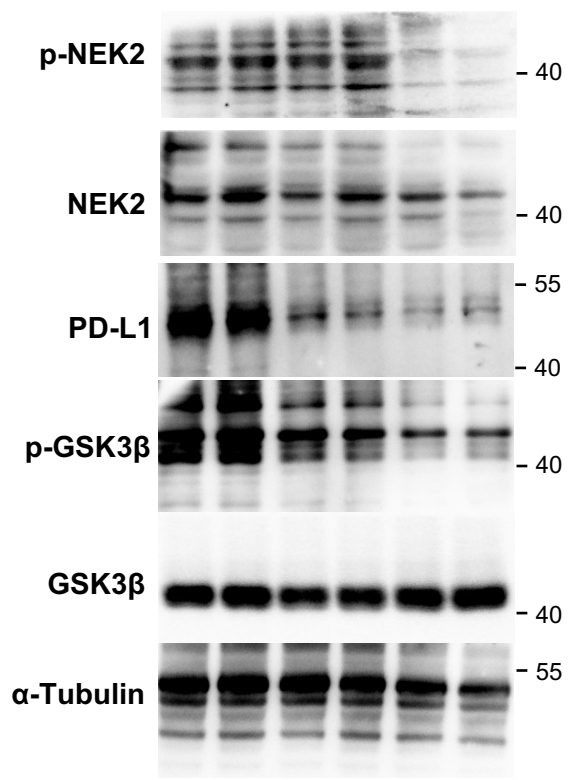

**Figure S9f**

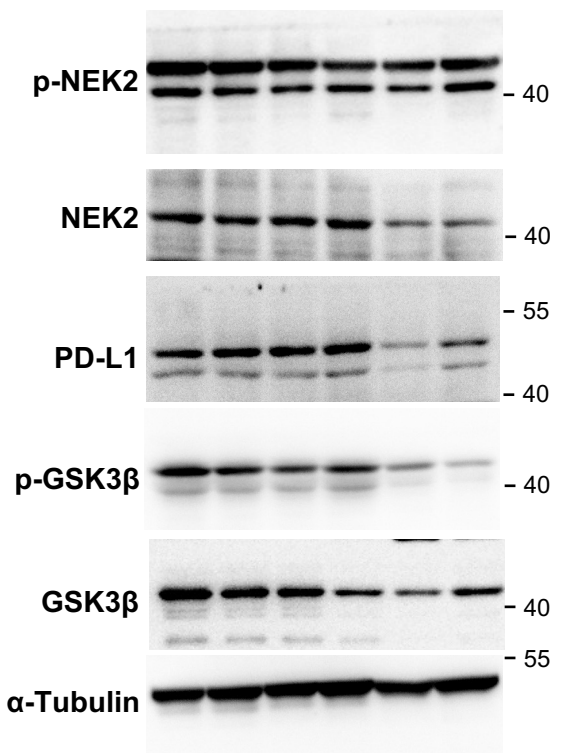

Supplement: Supplementary file 4 — Source data [file 41467_2021_24769_MOESM4_ESM.zip › Source data/Figure S9-uncropped gels.pdf]

**Figure S11e**

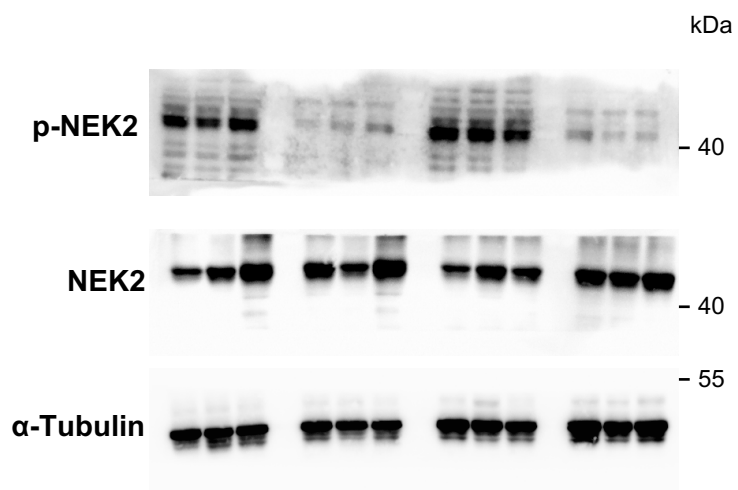

Supplement: Supplementary file 4 — Source data [file 41467_2021_24769_MOESM4_ESM.zip › Source data/Figure S13-uncropped gels.pdf]

**Figure S7b**

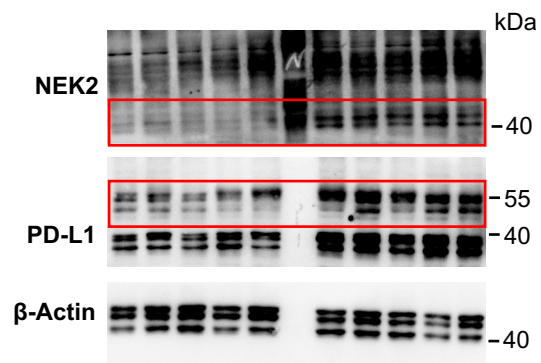

Supplement: Supplementary file 4 — Source data [file 41467_2021_24769_MOESM4_ESM.zip › Source data/Figure S7-uncropped gels.pdf]

Figure 6c

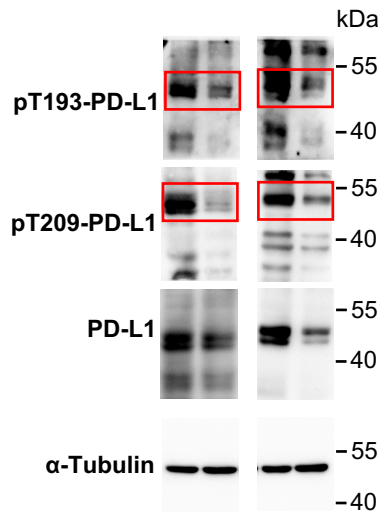

Figure 6d

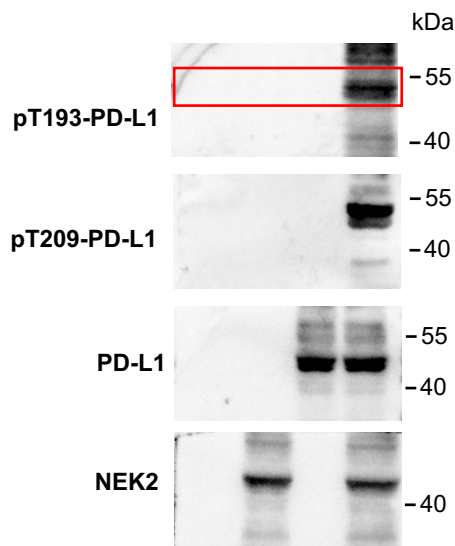

Figure 6g

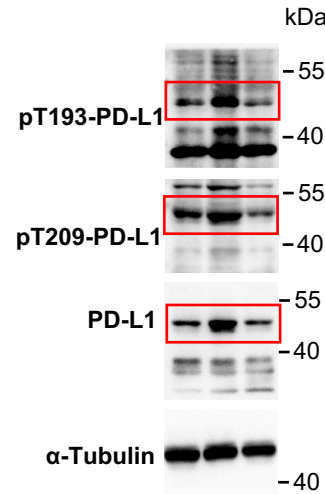

Figure 6e

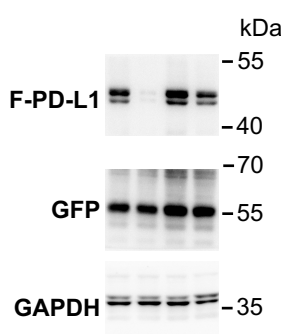

Figure 6h

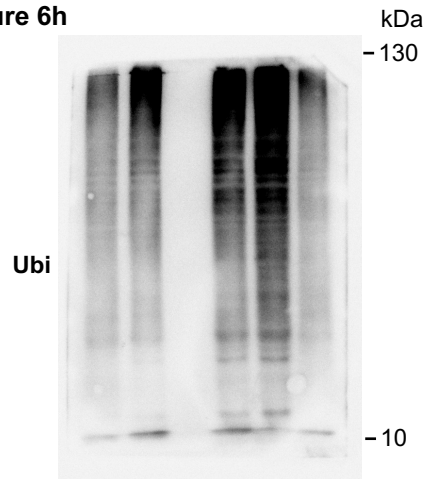

Figure 6i

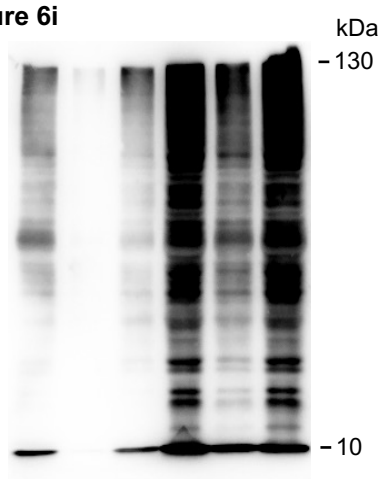

Figure 6f

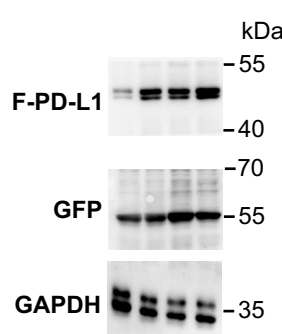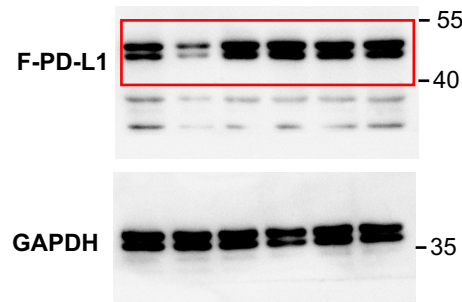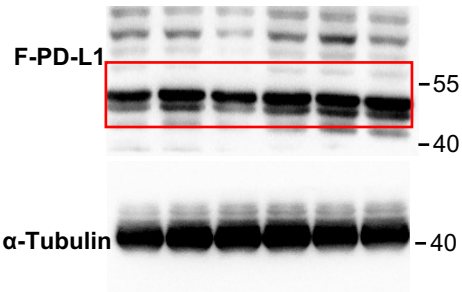

Supplement: Supplementary file 4 — Source data [file 41467_2021_24769_MOESM4_ESM.zip › Source data/Figure 6-uncropped gels.pdf]

Figure S6e

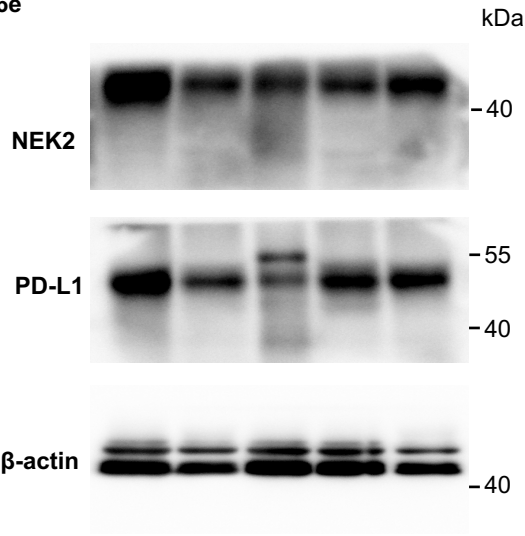

Supplement: Supplementary file 4 — Source data [file 41467_2021_24769_MOESM4_ESM.zip › Source data/Figure S6-uncropped gels.pdf]

Figure S9a

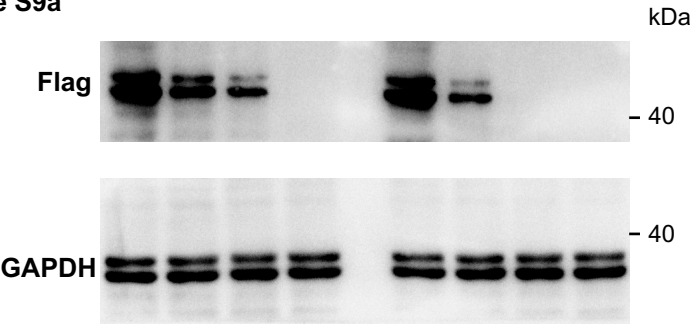

Figure S9c

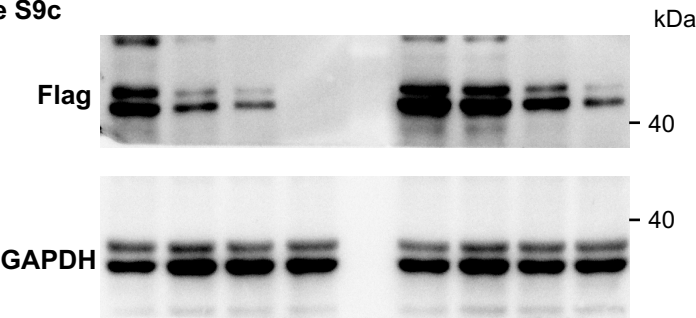

Figure S9e

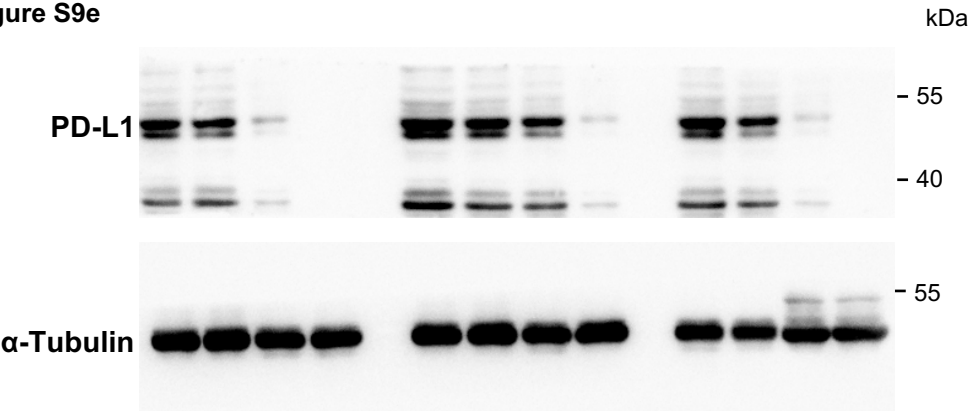

Supplement: Supplementary file 4 — Source data [file 41467_2021_24769_MOESM4_ESM.zip › Source data/Figure S11-uncropped gels.pdf]

Figure 4a

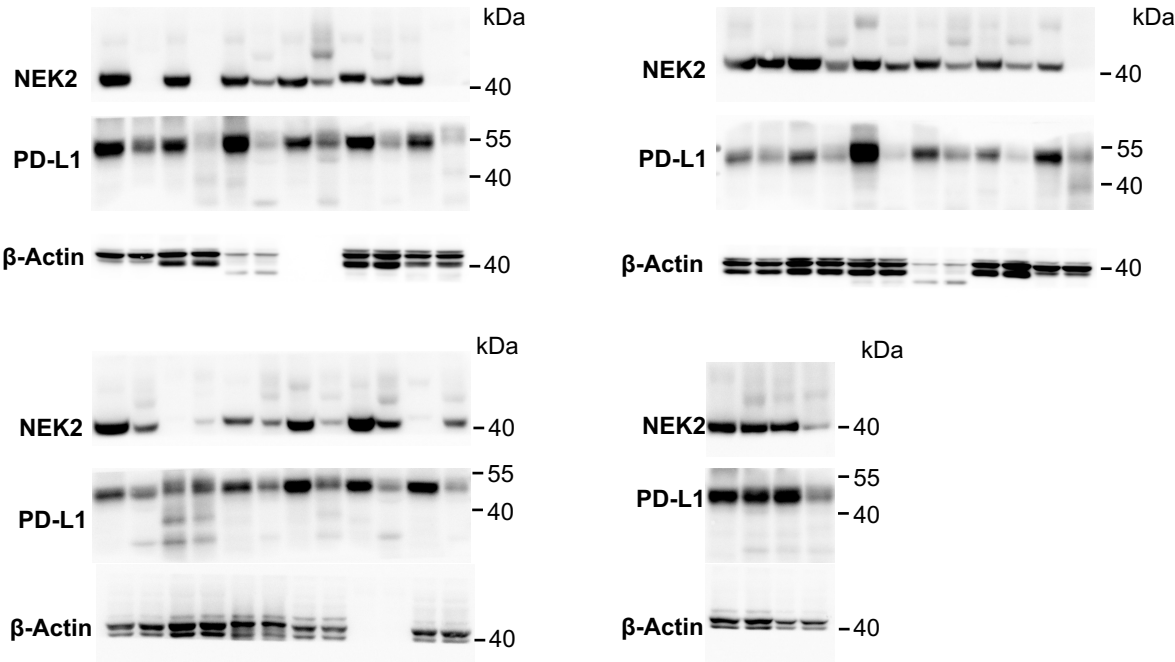

Figure 4e

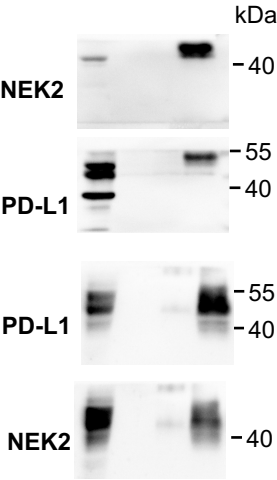

Figure 4f

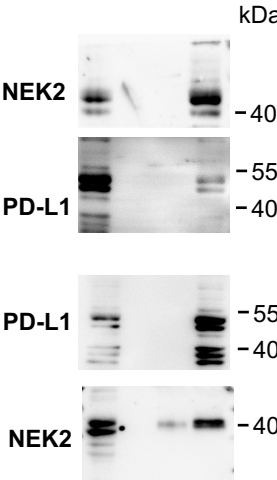

Figure 4g

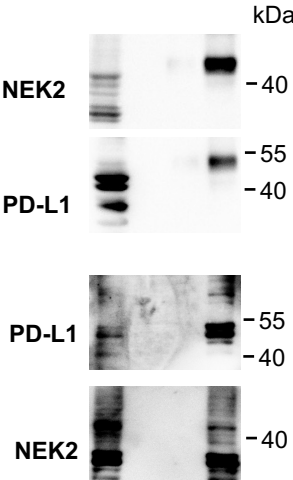

Figure 4h

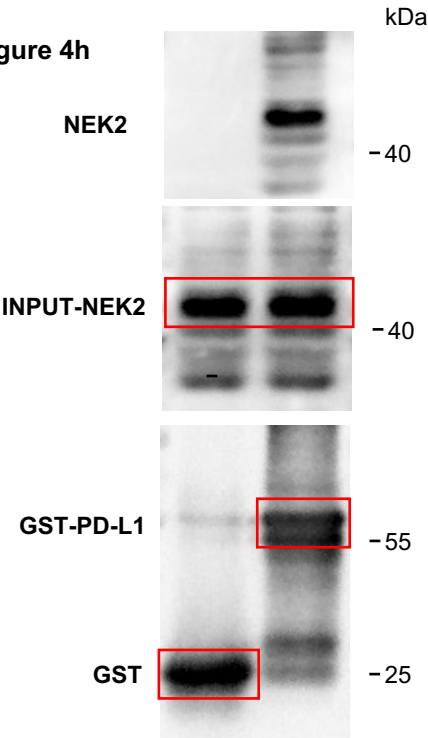

Supplement: Supplementary file 4 — Source data [file 41467_2021_24769_MOESM4_ESM.zip › Source data/Figure 4-uncropped gels.pdf]

**Figure S8a**

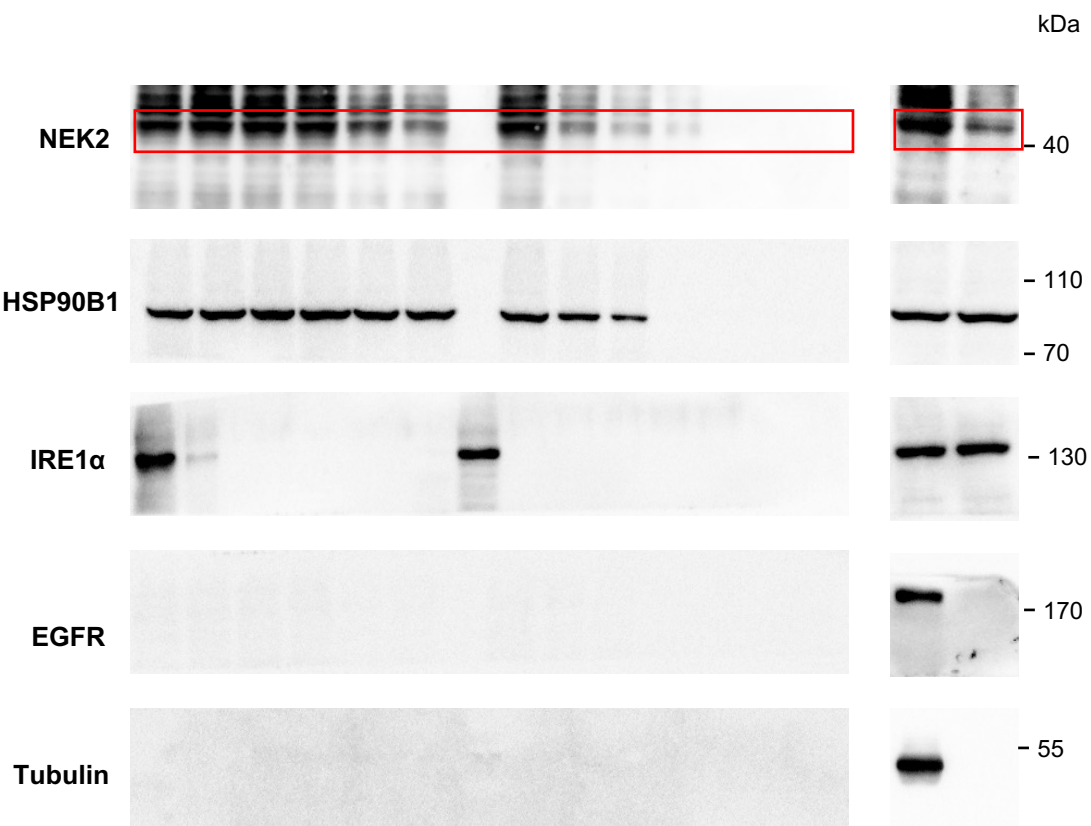

Supplement: Supplementary file 4 — Source data [file 41467_2021_24769_MOESM4_ESM.zip › Source data/Figure S8-uncropped gels.pdf]
